# Supplementary material for: Construction of ssDNA-Attached LR-Chimera Involving Z-DNA for ZBP1 Binding Analysis
Source: Molecules. 2022 Jun 9;27(12):3706. doi: 10.3390/molecules27123706 (PMC9230395; doi:10.3390/molecules27123706)
Supplement: Supplementary file 1 [file molecules-27-03706-s001.zip › molecules-1706324-supplementary.pdf]

SUPPORTING INFORMATION

For

Construction of ssDNA-attached LR-chimera involving Z-DNA for ZBP1 binding analysis

Lin Li[1], Ran An[1,2,*], and Xingguo Liang[1,2,*]

[1] College of Food Science and Engineering, Ocean University of China, Nucleic Acids Chemistry and Biotechnology Laboratory, No. 5 Yushan Road, Shinan-qu, Qingdao 266003, China; xbnllilin@163.com; ar@ouc.edu.cn; liangxg@ouc.edu.cn

[2.] Laboratory for Marine Drugs and Bioproducts, Qingdao National Laboratory for Marine Science and Technology, Qingdao 266235, China; ar@ouc.edu.cn; liangxg@ouc.edu.cn

[*] Correspondence: ar@ouc.edu.cn; Tel: +86-532-82031086; liangxg@ouc.edu.cn; Tel: +86-532-82031086

Table S1. The sequences used in this study

Name	Sequences（5′→3′）	Length (nt)
I-92a	CGTGTGATATAGGTGAACGCGCGTGTGTGTATATGTGTGC	40
I-92b	GATCGTAGAACTTGTGAACCCTTATACCATGCACGATCGCACATTTCGCACA	52
I-132a	GCACACATATACACACACGCGCGTTCACCTATATCACACGTGACTTGTAATTAGTAA CTACCAACGTGCG	70
I-132b	TGTGTGTCAGTCAGCGAAATGTGCGATCGTGCATGGTATAAGGGTTCACAAGTTCT ACGATC	62
L59	GACTGACACACACGCACGTACGTAGTTACTAATTACAAGACCTCACTATACTTACAT GT	59
L’49	ATAGTGAGGTCTTGTAATTAGTAACTACGTACGTGCGTGTGTGTCAGTC	49
I_n-92a	ATATGCCTAGTCTTCCATGACACTGAGACAACTGGAAACCACTCATGCGT	50
I_n-92b	AGATGCGAACTAGCACAGTGAACTCATCCACTAGAACTTGAC	42
Sp92a	GCATCTACGCAT	12
Sp92b	GCATATGTCAAG	12
I_n-132a	GAAGACTAGGCATATGTCAAGTTGACTTGTACTGTCTGGTAGTCAGCTAGGTAGTAC TGTTCTCTAGTGGATGA	74
I_n-132b	GTTCACTGTGCTAGTTCGCATCTACGCATGAGTGGTTTCCAGTTGTCTCAGTGTCAT G	58
Sp132a	TAGTCTTCCATGACAC	16
Sp132b	CAGTGAACTCATCCAC	16
I_B50	Biotin-TTTTTACCTCACTATACTTACATGTTTTTTGGTAGTTACTAATTACAAGT	50
I_{nB}50	Biotin-TTTTTACCTCACTATACTTACATGTTTTTTCTACCAGACAGTACAAGTCA	50

Note: The underline of L59 indicates 2 mismatches contained in the 41 bp complementary region of L59 and c132A.

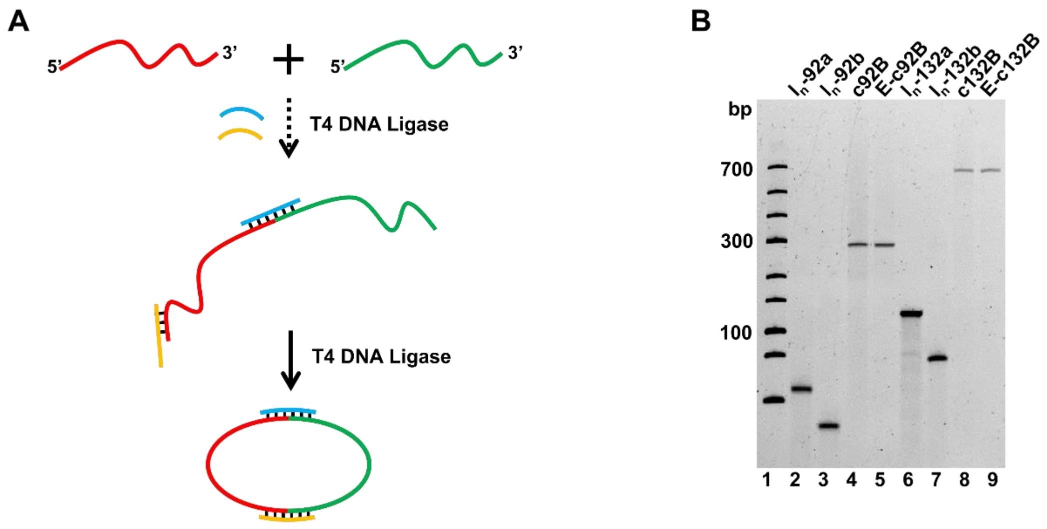

Figure S1. Preparation of circular ssDNA c92B and c132B with APP-poor sequences. **(A)** Preparation principle of one-pot circularization. Two splints and two fragments were mixed together to carry out ligation. **(B)** Electrophoresis analysis of circularization (8% dPAGE). Lanes 2 and 3: linear ssDNA fragments of l_n-92a and l_n-92b; lane 4: ligation product (c92B) of samples shown in lanes 2 and 3 at DNA concentration of 2 μM; lane 5: sample shown in lane 4 was digested by Exo I and Exo III; lanes 6 and 7: linear ssDNA fragments of l_n-132a and l_n-132b; lane 8: ligation product (c132B) of samples shown in lanes 6 and 7 at DNA concentration of 2 μM; lane 9: sample shown in lane 8 was digested by Exo I and Exo III. Other ligation conditions: c92B: [T4 DNA ligase buffer] = 0.05×, [T4 DNA ligase] = 0.25 U/μL, 37°C, 2 h; c132B: [T4 DNA ligase buffer] = 1×, [T4 DNA ligase] = 0.25 U/μL, 37°C, 2 h.

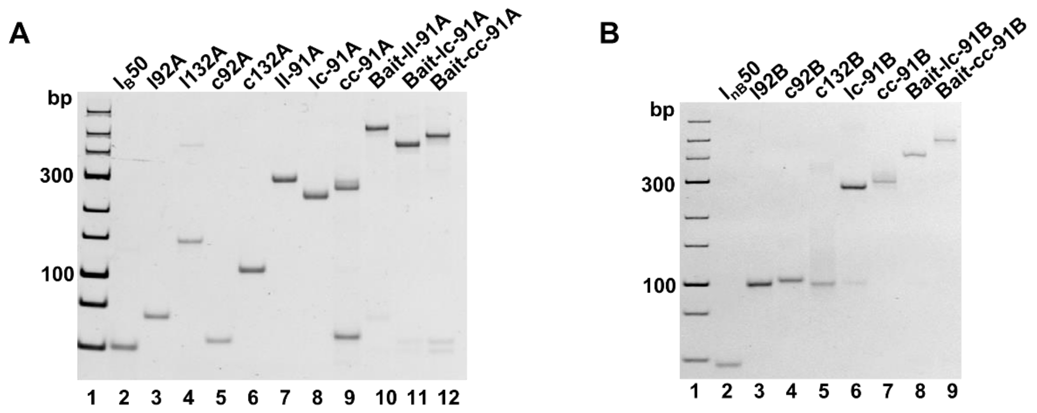

Figure S2. Preparation of Bait of LR-chimera for attaching to the biosensor. **(A)** Electrophoresis of Bait-cc-91A preparation (6% PAGE). Lanes 2-4: l_B50, l92A and l132A, respectively; lanes 5 and 6: c92A and c132A, respectively; lanes 7-9: hybrids of l92A and l132A (ll-91A), l92A and c132A (lc-91A), and c92A and c132A (cc-91A), respectively; lanes 10-12: hybrids of l_B50, l92A and l132A (Bait-ll-91A), l_B50, l92A and c132A (Bait-lc-91A), and l_B50, c92A andc132A (Bait-cc-91A), respectively. **(B)** Electrophoresis of Bait-cc-91B preparation (6% PAGE). Lanes 2 and 3: l_{nB}50 and l92B, respectively; lanes 4 and 5: c92B and c132B, respectively; lanes 6 and 7: hybrids of l92B and c132B (lc-91B), c92A and c132A (cc-91B), respectively; lanes 8 and 9: hybrids of l_B50, l92B and c132B (Bait-lc-91B), l_B50, c92B and c132B (Bait-cc-91B), respectively. All the gel were kept at 20°C during electrophoresis.
